# Supplementary material for: Are carbon emissions trading and green financial instruments synergistic? -Comprehensive quantitative research based on content analysis
Source: PLoS One. 2024 Mar 7;19(3):e0298601. doi: 10.1371/journal.pone.0298601 (PMC10919601; doi:10.1371/journal.pone.0298601)
Supplement: S1 Data — (ZIP) [file pone.0298601.s001.zip › Data/地级市Z_F工作报告.zip!/╝¬┴╓/═¿╗»╩╨/└┤╫╘╣1⁄2╓┌║┼í╛┬φ┐╦╩2╛▌═°í┐_2007.docx]

各位代表： 现在，我代表市政府，向大会作政府工作报告，请予审议。

　　一、2006年工作回顾

　　2006年是我市在“十五”的坚实基础上经济社会发展取得新突破的一年。市政府在市委的领导下，在市人大、市政协的监督支持下，带领全市广大干部群众，抓发展、抓重点、抓落实，实现了“十一五”良好开局。

　　过去的一年，全市经济快速发展。地区生产总值实现290.7亿元，比上年增长19.9%，高于全省增幅5.4个百分点。财政收入完成29.2亿元，增长21.5%，其中地方级财政收入13.3亿元，增长44.6%，高于全省增幅26.3个百分点。固定资产投资完成193.4亿元，增长74.2%，超过“十五”前四年的累计投资，高于全省增幅19.3个百分点。社会消费品零售总额实现106.2亿元，增长16.6%。城镇居民人均可支配收入10154元，增长15.3%。农民人均纯收入3700元，增长11.2%。

　　过去的一年，我市被命名为首批国家科技兴贸出口创新基地，被授予中国人居环境奖（水环境治理优秀范例城市），被评为中国旅游竞争力百强城市、中国百佳投资城市，被冠名中国通化葡萄酒城，通化在国内外的知名度进一步提升。

　　一年来，我们主要做了以下几方面工作：

　　（一）突出抓好招商引资和“项目建设年”活动。有针对性地走出去、请进来，与国内外350多户企业进行了洽谈对接，30多个层次较高的经贸考察团相继来访。全年落实招商引资项目1900项，到位资金150亿元，实际利用外资3350万美元。引进1000万元以上项目360项。同宁波华翔、北京中旺、苏宁集团、长春欧亚等20余户知名企业达成了合作协议。全市在建项目数量比上年增长1倍，新建项目增长1.2倍，实施亿元以上项目73项，其中10亿元以上项目5项。工业投资增量占全部投资增量的41.2%。通钢热轧、冷轧项目和500千伏输变电等一批关系长远发展的重大项目进展顺利。全力推进通化经济开发区建设，明确了发展规划及功能定位，完成了区划调整和机构设置，基础设施实现“五通一平”，配套面积达到5.2平方公里，26个1000万元以上项目落地开工。作为新的发展平台，通化经济开发区集聚工业项目的作用日益显现。

　　（二）狠抓工业提速增效和产业结构调整。规模以上工业实现产值313.8亿元，比上年增长30.2%；实现增加值114.6亿元，增长30.8%，高于全省增幅12.3个百分点，增速全省第二；实现利润16.6亿元，增长31%。规模以上工业企业发展到314户，新增64户。工业用电量34.2亿千瓦时，是历年来的最高值。高新技术企业发展到44户，居全省各市（州）第二位。建成了全国第一个国家级果酒及果蔬饮品质量监督检验中心。通化山葡萄酒获国家地理标识产品保护。修正、万通两件商标被认定为中国驰名商标。新创省级名牌产品20个。工业产销率达到96%，外贸出口增长96.4%。医药、冶金、食品三大支柱产业增加值增长31.8%，能源、新型建材、机械制造三个优势产业增加值增长29.4%。制定实施了围钢产业发展规划，开工围钢项目61项。接待海内外游客198万人次，实现旅游业收入12.8亿元，分别增长25%、28%。信息产业销售收入增长18.2%，10户企业、27种产品通过国家“双软认定”。

　　（三）扎实推进新农村建设和县域经济发展。落实了新农村建设总体规划和帮扶责任制，4个镇、14个村列入省级试点，71个村列入市级试点。实施“清洁文明工程”，村镇环境有新的改善，农村基础设施和社会事业发展步伐加快。新建农村水泥路534公里。全面完成了水利设施的年度建设任务。粮食生产再创历史最好水平，总产量达到159.2万吨。认真抓好“粮变肉”工程，标准化牧业小区发展到163个，重大动物疫病得到有效防控。累计82种农产品通过国家绿色标识认证。转移农村劳动力17.2万人。植树造林成效明显，实现了连续52年无重大森林火灾。农产品加工总量超过100万吨，省级以上重点龙头企业达到23户。与农发行吉林省分行签订了50亿元金融合作协议。全力抓好县（市）工业集中区建设，县域经济的发展活力明显增强。通化县、梅河口市进入全国最具投资潜力中小城市百强。

　　（四）加大基础设施和城市建设力度。市区绕越线暨单塔斜拉桥工程、梅河口市绕越线工程建成通车。通沈高速公路通化段全线开工。通丹、通长高速公路和通化机场项目列入国家规划，通丹铁路可研报告通过专家评审。组织实施城市棚户区改造，拆迁面积85.2万平方米，开工建筑面积123.6万平方米，交付使用面积47万平方米，7166户居民喜迁新居。我市被评为全省棚户区改造优秀城市，省政府在通化召开了回迁安置现场会。滨江西路、光明立交桥改造、江南新区路网、垃圾处理场等年初确定的城建重点工程全面完成，城市形象和环境设施进一步改善。精心实施绿化亮化工程，市区新增绿地3.1万平方米，滨江两岸亮化初见成效。加快供水供气供热改造，解决了市区周边及高层4.4万人的吃水问题和4000户居民的用气问题，新增供热面积80万平方米。启动了创建国家级卫生城工作，加强了市容市貌的整治和管理。污染治理、矿业整顿以及哈泥河湿地和饮用水源的保护等工作取得新进展。市区大气环境质量优良天数达到290天。

　　（五）进一步深化各项改革。解决了35户国有工业企业的改制遗留问题，对58户非工业国有企业进行了改制重组。推动改制企业发展，促进全民创业，民营经济增加值增长20.2%、纳税额增长19.6%。与深交所签订了中小企业上市培育工作协议，紫鑫药业通过了国家证监会的上市审核。深化农村综合配套改革，乡镇机构、农村教育和县乡财政新的管理体制和运行机制初步形成。清理整顿土地市场，经营性用地实行招标、拍卖和挂牌出让，加大了市本级收储土地的力度。城市信用社改制工作基本完成。调整和理顺了市对区的财政管理体制。实施了机关事业单位工资收入分配制度改革。规范行政事业性收费管理办法，明确了收支两条线及给予政策的程序。事业单位改革进展顺利。文化体制改革稳步推进。

　　（六）加快发展各项社会事业。启动了建设创新型通化的各项工作。列入国家和省科技发展专项27项。调整教育布局，深化教育改革，基础教育、职业教育加快发展。改造农村危旧校舍5.5万平方米。186所学校实现宽带联通。实施了高技能人才培养等人才工作重点项目。繁荣文艺创作，节日文化、广场文化丰富多彩。自安山城等3处遗址列入国家文物保护单位。广泛开展群众体育活动，竞技体育获省级以上金牌104枚。扎实推进新型农村合作医疗试点，惠及农民69万人。深化城市医疗体制改革，加强了公共卫生服务和疾病预防控制工作。广播、电视覆盖率分别达到96.5%、97.6%。计划生育“双管双助”经验在全国全军推广。农业普查工作进展顺利。市青少年宫、广电中心、中心医院外科楼等工程基本建成，师院分院、职教中心、市医院门诊楼、卫校易地改造等工程全面完工。加强公民思想道德建设，开展精神文明创建活动。妇女儿童、老龄和残疾人事业实现新发展。“五边”建设试点、“双拥”、“双支”工作取得新成绩。

　　（七）认真解决关系民生的问题。开发城镇就业岗位6.5万个，其中政府出资开发公益性岗位6149个，城镇新增就业5.4万人。对灵活就业人员实行了社保补贴政策。提高了最低工资标准、企业离退休人员养老金和城乡低保补差标准。养老和失业保险参保人数分别达到23.7万人和19.8万人，医疗、工伤、生育保险参保人数达到50.7万人。有18.2万人得到社会救助。扶贫助学3.2万人，其中享受“两免一补”的农村学生2.9万人。启动了城镇居民基本医疗保险。市区低保对象全部纳入大病医疗保险。新建、改造农村福利服务中心和敬老院57所。实施3年的联乡包村万户脱贫工程如期完成，188个贫困村和1万户贫困户基本脱贫。强化食品药品和市场秩序监管，加强安全生产工作，深入开展“平安通化”创建活动，建立突发事件应急机制，积极解决信访突出问题，维护了社会稳定。

　　（八）加强民主法制建设和政府自身建设。认真执行人大各项决议，主动接受人大的法律监督、工作监督和政协的民主监督。办理人大代表建议241件、政协提案206件。“五五”普法工作全面展开。基层民主进一步发展，社区建设得到加强。深入推行行政执法责任制，建立并落实了行政审批全程代办制、限时制和行政不作为问责办法。开展了治理商业贿赂等专项工作，廉政建设取得新的进展。加大软环境建设力度，设立了重点监测单位，查处涉软案件90件。严明工作纪律，规范了机关办事程序。各级公务员的精神状态和服务意识有新的转变，求发展、谋发展的热情高、干劲足、势头好。

　　各位代表，过去的一年，振兴通化老工业基地的各项工作全面推进，主要经济指标实现大幅度增长，重点工作取得新的突破。这些成绩，是市委正确领导的结果，是市人大、市政协有效监督、大力支持的结果，凝聚着全市人民的智慧和汗水。在此，我代表市政府，向广大干部群众，向人大代表、政协委员，向各民主党派、各人民团体和社会各界人士，向驻通国省属单位和驻通部队、武警官兵，向所有关心和支持通化发展的同志们、朋友们，致以崇高的敬意和衷心的感谢！

　　我们也清醒地认识到，我市经济社会发展还存在不少问题：经济总量不大，综合实力不强；工业规模偏小，产业竞争力较弱；对外开放度不够，基础设施建设还较滞后；农村生产力水平较低，农民持续增收难度增加；扩大就业和社会保障压力增大，群众的生产生活方面还有许多难点问题没有解决好；安全生产形势比较严峻，影响社会稳定的因素依然存在；软环境建设还有差距，政府机关的服务和业务水平有待改进，等等。我们一定高度重视这些问题，认真加以解决。

　　二、2007年主要任务

　　市第五次党代会绘就了未来五年的发展蓝图，提出了建设富强和谐通化的目标要求。我们要承担起新的历史责任，抓住新的发展机遇，集中精力抓发展、抓重点、抓落实，为建设经济更好更快发展的通化、群众生活更加富裕的通化、人居环境更加优美的通化、社会更加和谐稳定的通化努力工作。

　　2007年，市政府要坚持以科学发展观为统领，全面落实市第五次党代会精神，紧紧围绕振兴通化老工业基地和构建和谐社会中心任务，强基础、调结构、增效益，着力抓好项目建设，着力调整优化经济结构，着力推进改革开放和科技创新，着力加强基础设施建设、城市建设和新农村建设，着力加快社会事业发展，着力解决民生问题，提升工业化、城镇化和市场化水平，努力实现经济社会更好更快发展，开创建设富强和谐通化的新局面。

　　今年的主要预期目标：地区生产总值增长15%，财政收入与经济同步增长，万元GDP能耗下降6%，固定资产投资增长50%，社会消费品零售总额增长15%，城镇登记失业率控制在4.6%以内，城镇居民人均可支配收入增长10%，农民人均纯收入增长7%，人口自然增长率控制在3.8‰以内。

　　保持全市经济社会发展的好势头，全面完成今年的目标任务，必须从通化实际出发，坚定不移地走工业立市之路、投资拉动之路、开放带动之路、改革创新之路和可持续发展之路，努力做到好中求快、快中求进，为实现“十一五”地区生产总值、财政收入、工业总产值三个翻番奠定坚实基础。

　　（一）继续开展“项目建设年”活动，加大招商开放工作力度。举全市之力，进一步聚焦项目建设，推动投资、引资和对外开放取得新的突破。突出抓好一批关系全局的重大项目。全市固定资产投资必保完成290亿元，工业投资达到145亿元。实施亿元以上项目85项，其中5亿元以上项目14项。新增130万吨热轧、2680立方米高炉项目建成投产，100万吨冷轧项目实现部分产出。加快40万吨硅钢、200兆瓦热电联产、500千伏输变电等大项目的实施进度。全力推进开放通道建设，积极打造通化外围“2小时经济圈”。通沈高速公路完成路基、桥涵工程和70%的路面底基层，启动建设通丹、通长、梅沈高速公路和通丹铁路，进京列车争取夕发朝至。继续做好通集高速公路和通化机场等重大项目的前期工作。有效利用开发银行、进出口银行和商业银行的金融贷款，争取国债项目和资金。严把项目审核关，提高投资质量和效益。坚持把招商引资作为加快发展的重中之重，立足产业资源优势和外商投资需求，包装重点项目，组建招商小分队，灵活开展招商，着力引进有实力的战略投资者和知名企业集团特别是500强企业。加快国家科技兴贸出口创新基地和集安“四区一桥”口岸工程建设，引进和发展一批高成长性的出口大户，扩大对外贸易。加强企业管理体系和产品质量认证，支持企业开拓国内外市场。全年引进资金210亿元，实际利用外资3800万美元，引进1000万元以上项目455项，实现外贸进出口总额4.2亿美元。完善重大项目和招商开放工作协调机制，落实招商引资责任制和领导包保制度，严格考核，兑现奖惩。

　　（二）推动产业结构优化升级，促进工业提速增效。围绕建设工业主导型、生态效益型的新型产业基地，统筹规划“钢铁城”、“医药城”和“葡萄酒城”发展，加快吉林省冶金产业基地、现代中药及生物药产业基地和长白山特色食品产业基地建设。坚持支持通钢、服务通钢、发展通化的原则，为2008年通钢实现600万吨生产能力提供支持。大力发展围钢经济，延长钢铁产业链条，抓好上下游产品及配套项目，规划的97个围钢项目争取全部开工。推进医药工业、医药商贸、医药科研、医药教育、中药材基地“五位一体”协调发展，推动医药企业的整合重组和对外合资合作，开发生产具有自主知识产权的国家一二类新药，提高医药产业的集中度和整体竞争力。组织实施好长白山药谷规划。加大葡萄酒产业的结构调整和规范管理，扶大扶强重点骨干企业，发展振兴通化的葡萄酒品牌。深度开发优质农产品和长白山特产资源，加强项目包装和对外推介，建设好人参产业园、果仁食品工业园和食用酒精、鹅鸭肥肝等重点项目。加大优势产业的项目谋划，加快发展能源、新型建材、机械制造、汽车零部件产业。争取在我市设立国家石油机械质量监督检验中心。积极开发油页岩、煤炭、铁矿、水能等资源和林木生物质能源。在调整优化产业结构的同时，全力推进通钢、修正等重点骨干企业和现有工业项目的产能释放，挖掘改制企业的增长潜力，扶持成长性强的企业快速发展，评选工业企业30强，努力实现工业提速增效。加快实施技术创新“双百”工程，着力建设100个标志性工业项目，竣工项目的产能达到70%以上。深入实施品牌战略，培育一批国家和省级名牌产品。发展循环经济和节约型产业，抓好重点行业和企业的降耗减排工作。加强生产运行组织，搞好煤电油运等要素衔接。规模以上工业增加值增长18%、利润增长12%以上；新增规模以上工业企业40户以上。

　　（三）加速通化经济开发区建设，进一步拓展发展空间。突出重大项目和产业集群的招商引资，加快开发区的基础设施建设和项目摆放，在引进和建设超亿元的大项目上实现突破性进展。全面推进机械冶金、生物医药、有机食品3个工业园区的基础设施建设，完成自安、张家园区“七通一平”工程，为项目和企业集聚搭建平台。支持具备条件的市区工业企业通过置换重组，退城进区，集约发展。严把入区项目的环保关、技术关，提高单位土地面积的投资强度和产出效益。创新管理和服务，使开发区更具活力和吸引力。全年开发区引进1000万元以上项目30项，去年开工的项目力争多数投产。

　　（四）加强城市建设与管理，增强城市综合服务功能。引进先进的规划理念，修编城市总体规划，重点编制中心城区、江南新区和浑江两岸的控制性详规，提升城市设计和建筑品位，建设更加适宜人居和创业的山水城市。严格土地规划管理，加大收储力度，集约节约利用土地。刚性执行城市规划，维护规划的权威性。实施10项城建重点工程。一是城市棚户区改造工程。拆迁改造市中心区域棚户区，完成拆迁面积65万平方米，开工建筑面积100万平方米，保证开发质量和回迁安置，力争提前一年完成改造任务。二是城市街路建设工程。维修和改造龙泉路、玉泉路等20条主次街路和巷路，进一步完善城区路网。对光复路及周边区域进行全面改造，建设特色街区。三是江南新区建设工程。规划实施路网和水气热等基础设施及部分开发改造工程，推动江南整体开发。四是沿江景观带工程。对浑江两岸统一进行景观设计，建设滨江西路带状公园，改扩建玉皇山桥至一期平湖路段，新建沿江绿带、观景平台、亲水休闲区和健身活动区。五是绿化亮化工程。突出主要街路和居民小区，加大绿化美化力度，新增绿地面积7.5万平方米，争创省级园林城市。对浑江两岸及周边公建楼和城区街路巷路进行亮化升级和照明设施改造。六是供水供气供热改造工程。改造供水设施，解决高层区域的3万人吃水问题。完成第二煤气储配站续建工程，解决3000户居民用气问题。继续实施老旧楼供热管网改造。七是环卫设施建设工程。新建污水处理站，完善压缩垃圾中转楼等配套设施，使垃圾处理工程全面投入使用。启动建设10万吨城市污水处理项目。八是市水泥厂开发改造工程。拆迁市水泥厂，消除城市污染源，对该区域进行规划建设和综合整治，改造城市出城口，改善周边环境。九是玉皇山公园景区改造工程。对玉皇山公园进行统一规划设计，分期改造。突出城市景观和百姓休闲健身功能，注重人与自然生态的和谐，加强公园管理和建设。十是规划设计集中供热三期、二道河整治和第二水厂等重点工程，力争早日开工。与此同时，深入开展创建国家级卫生城活动，认真解决市民关注的城市管理薄弱环节。综合整治市容市貌、环境卫生和市场秩序，提高城市交通的管理和建设水平。加大环境保护力度，加强饮用水源、重点流域的污染治理和生态建设。争取哈泥河湿地晋升为国家级自然保护区。市区大气环境质量优良天数超过295天。

　　（五）发展壮大县域经济，抓好社会主义新农村建设。深入实施县域突破战略，各县（市、区）要争先晋位、加快发展。全面推进县（市）工业集中区建设，完善基础设施和服务功能，集中摆放工业项目。建设好全省首批启动的4个农业产业化集中区，加强对龙头企业的扶持和重点项目的谋划，农产品加工转化能力达到115万吨、实现销售收入100亿元。优化种植结构，加强优质水稻、特用玉米、优质大豆基地建设，粮食总产稳定在150万吨左右。优先发展畜牧业，推进畜产品精深加工，抓好畜禽优质品种培育、引进和疫病防控，促进畜牧业全面升级。加强森林资源保护，发展林业经济和林产品加工业。完善农业科技创新体系，推广应用一批先进适用技术。发展农村新型合作组织，建设农村市场网络。加强组织，搞好培训，转移农村富余劳动力18万人，大幅度增加劳务经济对农民收入增长的贡献率。把发展农村经济、改善人居环境、促进农民增收作为新农村建设的中心任务，加大产业项目、基础设施和公共事业投入，重点抓好农村道路、水电、通讯以及教育、卫生、文化等设施建设，有规划有步骤地搞好村屯环境综合整治。总结新农村建设试点经验，积极探索建设模式。加强县城和中心镇建设，逐步完善城镇功能。启动县（市）棚户区改造。

　　（六）推进服务业跨越式发展，培育新的经济增长源。制定服务业发展跨越计划，提升服务业整体水平。服务业增加值增长15.5% 。突出发展生产性服务业，培育和引进现代物流龙头企业，重点建设市区、梅河口、快大物流园区和开发区等专业物流市场。加快发展连锁经营、物流配送、电子商务和金融、保险、咨询、中介等新型业态和服务方式。建设“数字通化”平台，培育发展信息服务业。充实提升商贸流通、餐饮娱乐、社区服务、物业管理等生活性服务业。加强与国内外知名商贸企业的合资合作，积极推进传统商业街区和江南新区的整体改造，筹建具有区域辐射作用的大型商贸服务中心和特色街区。活跃住房二三级市场，促进房地产业健康发展。启动争创中国优秀旅游城市工作，开发推介古迹、生态、冰雪、民俗、边境和红色之旅等旅游品牌，整合推出具有通化特色的休闲避暑度假旅游项目。筹建高句丽文化旅游开发区，加快五女峰、龙湾、白鸡腰、三仙夹、石湖国家森林公园及罗通山等重点景区建设。规划建设通化滑雪场，拓展冰雪休闲运动项目。旅游业收入增长30%。

　　（七）实施民营经济腾飞计划，增强经济发展活力。把民营经济摆上更加突出的位置，推动全民创业，营造崇尚创业、重商致富的社会氛围。出台并认真落实具体的鼓励政策措施，创造良好环境，大力培育发展个体工商户和中小企业。实行“非禁即入”，破解制约民营经济发展的各种障碍。引导民营经济围绕支柱优势产业和大企业开展协作配套和深加工。启动100户民营企业融资支持计划，搭建中小企业融资平台。完善信用担保体系，担保能力达到1.8亿元。发挥创业大厦服务功能，加强创业辅导，建立中小企业创业基地和服务平台，设立创业培训专项资金。加强民营企业家培养，实施服务民营企业项目计划。鼓励机关、事业单位人员领办创办实业，选树和重奖“创业功臣”。制定完善指标体系，推动民营经济加快发展。民营经济主营业务收入增长30%以上。

　　（八）继续深化改革，加快体制机制创新。巩固国企改革成果，抓好改制企业新机制的建立和运营。扩大非工国企改革范围，全市现有的193户非工企业全部制定改制方案并进入操作程序。实施好列入国家政策性破产和依法破产项目，做好通钢分离办社会职能工作。启动厂办大集体企业改革。深化投资体制改革，推行预算内项目“代建制”。强化对股份制企业的监管和服务，争取上市融资，保护股民权益。加强金融生态环境建设，筹建地方商业银行。完善公共财政制度，加强税收征管，确保财政收入稳定增长。推行国库集中支付制度改革，提高重点支出保障能力。完成事业单位人事制度改革任务。积极推进文化体制改革。进一步搞好农村综合改革，实施集体林权制度改革，做好化解乡村债务试点工作。开展农业保险试点。

　　（九）坚持科教兴市和人才兴业，协调发展各项社会事业。抓好中药创新药物开发等重大科技专项和10项产学研攻关项目，加快自主创新和引进消化吸收再创新。启动全民科学素质行动计划。加强创新型和创业型人才的培养、引进和使用，做好各类人才的培训工作。深化教育教学改革，巩固基础教育成果，全面实施素质教育。加大农村中小学控辍力度。大力发展职业教育，整合高等教育资源，筹建通化职业技术学院，培养急需的技能型人才。支持通化师院加快发展。繁荣文艺创作，抓好基层文化建设，办好各种文化节庆活动。加大社会事业投入，建成使用市青少年宫、广电中心，规划建设市档案馆和体育休闲健身中心，完成高句丽遗迹保护二期工程、万发遗址博物馆、靖宇陵园配套工程和市中心医院扩建工程。谋划和扶持一批文化产业重点项目。支持英语辅导报做大做强。完成广播电视“村村通”工程，发展数字电视用户2万户。落实全民健身计划，备战第十一届全国冬运会。加强农村三级医疗卫生服务网络和城市社区卫生服务机构建设，健全疾病预防控制和医疗救治体系。稳定低生育水平，提高出生人口素质。树立社会主义荣辱观，加强精神文明建设，培育文明道德风尚。强化未成年人思想道德建设。保障妇女儿童权益。发展老龄和残疾人事业。推动民族团结进步。加强国防教育，深入做好国防动员。创新“双拥”、“双支”工作，争创全国双拥模范城“六连冠”。

　　（十）高度重视民生问题，维护社会和谐稳定。加大民生问题解决力度，让人民群众共享改革发展成果。一是努力扩大就业。开发城镇就业岗位5.6万个，城镇新增就业4.5万人。建立“零就业”家庭动态援助机制，全部解决“零就业”家庭的就业问题，完成政府出资三年购买1万个公益性就业岗位任务，实施低保家庭适龄子女就业技能培训计划。建成使用市人力资源市场。二是加强社会保障工作。抓好非公经济组织和灵活就业人员的参保工作，新增养老、失业、医疗、工伤、生育保险参保人数10万人。加大保费征缴力度，确保按时足额发放。提高公益性岗位工资和失业保障金补贴标准。出台失地农民的社会保障制度和养老保险办法。三是抓好社会救助工作。完善社会救助体系，发展社会福利和慈善事业，切实解决困难群众的就业、就学、就医、住房等实际困难。完成农村福利服务中心建设任务。四是实现城乡最低生活保障全覆盖。全面落实低保补差标准，城市月人均达到85元，农村年人均由180元提高到360元，对特殊困难低保家庭适当提高补助标准。五是建立完善城乡居民医疗保险制度。全面推行城镇居民基本医疗保险，对困难群众和老年人、学生等群体给予参保缴费补贴。建立覆盖所有县（市）的新型农村合作医疗制度。六是完善农村义务教育经费保障机制。全部免除农村义务教育阶段的学杂费。加强城乡扶贫助学，不让困难家庭的学生因贫困而失学。七是实施农村饮水安全工程。建成农村饮水安全工程50处，解决6.4万人和1.9万头大牲畜的饮水安全问题。全部解决农村饮用高氟高砷水问题。八是加强食品药品和餐饮卫生监管。以粮食、蔬菜、生肉、海产品、熟肉制品和药品流通市场为重点，加大执法力度和检验监测体系建设，让群众吃上放心食品、用上放心药品。九是保持社会稳定。严格落实安全生产责任制，防止重特大事故发生。加强应急管理，完善预案体系，妥善处置突发公共事件。加强“五边”建设，维护边境安全稳定。加强社会治安综合治理，抓好公安队伍和派出所建设，严厉打击各种犯罪，创建“平安通化”，确保人民群众安居乐业。

　　三、加强政府自身建设

　　推进管理创新，倡导勤奋好学、学以致用，心系群众、服务人民，真抓实干、务求实效，艰苦奋斗、勤俭节约，顾全大局、令行禁止，发扬民主、团结共事，秉公用权、廉洁从政，生活正派、情趣健康等八种良好风气，提高政府的公信力和执行力，建设服务政府、法治政府、责任政府和效能政府。

　　坚持勤政为民，强化服务意识。把实现群众的愿望、满足群众的需要、改善群众的生活作为政府工作的出发点和落脚点，从群众最关心、最直接、最现实的利益问题入手，尽职尽责地为群众办实事、办好事。对确定的城建重点工程、关系民生的重点工作，要集中力量、集中资金，精心组织实施，确保全面落实。创新服务方式，为企业和群众提供优质高效服务。做好信访和市长公开电话工作，为群众排忧解难。加快发展电子政务，推进公共服务信息化。落实市民旁听、决策听证、新闻发布、社会公示等制度，保障群众的知情权、参与权和监督权。

　　转变政府职能，大力改进政风。按照建立和完善社会主义市场经济体制的要求，规范政府职能定位，认真履行经济调节、市场监管、社会管理和公共服务职能。开展政府绩效评估，落实工作责任制，提高行政效能和工作效率。大兴求真务实和创新之风，多干群众急需和受益的事情，多干打基础和利长远的事情。改进会风和文风，提倡少开会、开短会，集中精力抓落实、抓实施。坚决不做脱离实际和违背群众意愿的事情，坚决从文山会海、迎来送往中摆脱出来，坚决克服形式主义、防止弄虚作假。注重工作实绩，鼓励干事的，支持干事的，表彰干事的。加强对公务员的教育、管理和监督，建设高素质的公务员队伍。全体公务员要学经济、懂经济，勤勉尽责，勇于创新，争创一流业绩。

　　严格依法行政，科学民主决策。贯彻《全面推进依法行政实施纲要》，按照法定权限和程序履行职责。落实行政问责制、执法责任制和过错追究制，规范行政执法行为，公正执法、文明执法。自觉接受法律监督、民主监督和社会监督，认真执行市人大常委会的决议，坚持重大事项向市人大常委会报告和向市政协通报制度，及时办理落实人大代表建议和政协提案。深入开展“五五”普法教育。完善公众参与、专家咨询论证和政府决策相结合的民主决策机制，充分听取人大代表、政协委员、各民主党派、人民团体以及群众的意见和建议，确保决策正确有效。

　　优化发展环境，加强廉政建设。深化政务公开，积极筹建政务大厅，全面推行行政审批权相对集中改革，确保行政权力在阳光下运行。建立健全重大招商引资项目行政审批全程代办制和限时制。继续开展民主评议软环境、政行风和重点岗位负责人活动，发挥行政投诉举报中心作用，严肃查处涉软案件。加强政务诚信、商务诚信、社会诚信建设。坚持从严治政，抓好惩治和预防腐败体系建设，加强行政监察、审计监督，纠正部门和行业不正之风，坚决惩治腐败。深入推进治理商业贿赂工作。创造风清气正的行政环境，建设人民群众满意的政府机关。

　　各位代表！振兴通化老工业基地，建设富强和谐的通化，是时代赋予我们的光荣使命。我们充满信心，同时也深感责任重大。我们要在市委的领导下，在市人大、市政协的大力支持下，团结和带领全市人民，抢抓机遇，开拓进取，扎实工作，为圆满完成今年的目标任务，为实现通化的更好更快发展而努力奋斗！
